# Gauging the Impact of Cancer Treatment Modalities on Circulating Tumor Cells (CTCs)

**DOI:** 10.3390/cancers12030743

**Published:** 2020-03-21

**Authors:** Trevor J. Mathias, Katarina T. Chang, Stuart S. Martin, Michele I. Vitolo

**Affiliations:** 1 Program in Molecular Medicine, University of Maryland Graduate Program in Life Sciences, Baltimore, MD 21201, USA; Trevor.Mathias@som.umaryland.edu (T.J.M.); ktchang@som.umaryland.edu (K.T.C.); 2 Department of Physiology, University of Maryland School of Medicine, Baltimore, MD 21201, USA; ssmartin@som.umaryland.edu; 3 Stewart Greenebaum NCI Comprehensive Cancer Center, University of Maryland School of Medicine, Baltimore, MD 21201, USA

**Keywords:** cancer, metastasis, circulating tumor cells, CTCs, dissemination, chemotherapy, radiotherapy, surgery resection

## Abstract

The metastatic cascade consists of multiple complex steps, but the belief that it is a linear process is diminishing. In order to metastasize, cells must enter the blood vessels or body cavities (depending on the cancer type) via active or passive mechanisms. Once in the bloodstream and/or lymphatics, these cancer cells are now termed circulating tumor cells (CTCs). CTC numbers as well as CTC clusters have been used as a prognostic marker with higher numbers of CTCs and/or CTC clusters correlating with an unfavorable prognosis. However, we have very limited knowledge about CTC biology, including which of these cells are ultimately responsible for overt metastatic growth, but due to the fact that higher numbers of CTCs correlate with a worse prognosis; it would seem appropriate to either limit CTCs and/or their dissemination. Here, we will discuss the different cancer treatments which may inadvertently promote the mobilization of CTCs and potential CTC therapies to decrease metastasis.

## 1. Introduction

For a majority of cancers, the 5-year survival rate of patients who present with localized or regional disease is steadily trending upwards [1] (US National Cancer Institute Surveillance, Epidemiology, and End Result (SEER) registries—data from 1995–2000 and 2004–2020 collected in 2005 and 2015 respectively). However, upon development of metastatic disease, not only do survival percentages plummet, but there has not been any increase, and in many cancer subtypes a decrease, in the 5-year survival rates for most metastatic cancers (breast, bladder, melanoma, ovarian, pancreatic, prostate, cervical, uterine). The few exceptions are colorectal, esophageal, lung, and oral cancer metastases, where there is only a very minimal increase in 5-year survival (mostly less than 3%) [1] (US SEER registries—data from 1995–2000 and 2004–2020 collected in 2005 and 2015 respectively). This is likely due to the focus of both drug development and clinical efficacy studies on inhibition and regression of tumor cell growth which largely ignores circulating or disseminated tumor cells, which could be an important, yet an under-examined phase of the metastatic cascade. In light of recent studies showing that radiotherapy, surgery and chemotherapy have the potential to dramatically increase levels of tumor cells in the bloodstream even if the primary tumor is successfully removed or destroyed [2,3], understanding the complex biology of CTC dissemination is paramount. 

## 2. CTCs and Metastatic Efficiency

**Figure 1:**
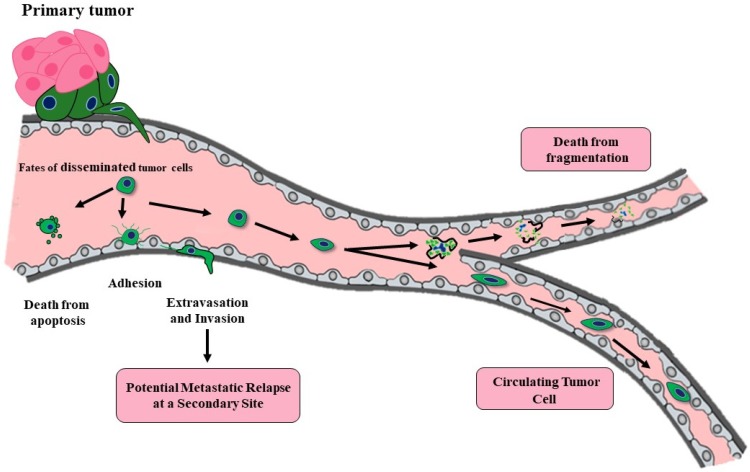
Tumor cell dissemination. Once tumor cells have entered the bloodstream, they may die due to apoptosis, sheer stress, or fragmentation when being pushed through tiny capillaries. In the minor cases that circulating tumor cells (CTCs) are viable and survive, they may be able to continue the metastatic process. At which point, they would need to attach to the endothelial cell wall, extravasate, and invade into the surrounding tissue where they may lay dormant for years before becoming an overt micrometastasis. Individual CTCs do not remain in the bloodstream long (~10–15min at the most) before they reach the closes capillary bed.

By the time a primary cancer mass is clinically detectable, it contains a minimum of 10^7^–10^8^ cells (approximately the size of 0.5 cm^3^) [4,5]. Tumors may only reach this size and continue their growth by promoting new and incorporating current vasculature via angiogenesis [6]. The resulting vessels provide nutrients for the growing tumor as well as access for mobilized tumor cells. Even a small primary tumor with neovasculature can “shed” millions of tumor cells daily into the bloodstream by the time of first detection. Over 40 years ago, Butler and Gullino determined that 1.7–4.6 × 10^6^ tumor cells were shed every 24 hour per gram from growing hormone dependent rat mammary tumors [7]. However, although tumors may release millions of CTCs daily, a vast majority die due to the inhospitable environment within the vasculature. Thus, hematological metastasis is a highly inefficient process (Figure 1). Any hematologic disseminated CTCs (at this point, more correctly termed disseminated tumor cells or DTCs) need to be able to survive hemodynamic stress and trapping within capillaries, need to adhere to the endothelial cell wall and extravasate into the surrounding tissue with a potential suitable metastatic niche where they may remain dormant. The potential result of hematologic dissemination is distant metastases, opposed to local metastasis to the lymph nodes which likely occurs through the lymphatics. DTCs may be able to initiate growth to form a micrometastases, but then these microscopic secondary tumors need to recruit new and existing vasculature once again in order to continue grow into a clinically relevant macrometastases [8]. The percent efficiency of CTCs able to metastasize is likely dependent on cancer type, mutations, immune system evasion, and metastatic niche requirements. An early study aimed to determine metastatic efficiency of CTCs was performed by Fidler [9]. Labeled B16 melanoma cells were injected (2 × 10^5^) into the tail vein of mice and after 14 days, an average of only 400 cells remained in the lungs. Additionally, an average of 78 pleural metastases (<0.04%) and a lesser unknown number of interior parenchymal metastases occur presumably in mice allowed to live past 14 days. Unfortunately, it is unknown whether these are clinically relevant micrometastases, especially since the majority of micrometastases are not likely to progress to overt metastases. Luzzi et al. [10] used a similar model of metastasis, injecting fluorescently labeled B16 melanoma cells into the superior mesenteric vein and determined that 2% of the injected cells formed micrometastases in the liver, only 1 out of 100 of these micrometastases would go on to form a macroscopic tumor (~0.02% of injected cells). Both studies employed highly metastatic B16 melanoma cells. It is likely that the metastatic efficiency is lower for other cancers. Supporting this notion is a modelling study completed using a large cohort of patients with breast cancer which determined that only 1 CTC from every 60 million cells escaping from the primary tumor has the ability to metastasize [11] which equated to less than 0.000002%. The good news is that the metastatic process is highly inefficient, but the not so good news is that tumors may be shedding large numbers of CTCs very early during their development. It is very possible that at the time of regional or even local diagnosis, tumors cells have already disseminated, and may be existing below the levels of clinical detection as either single cell or micrometastases. Other studies which analyzed expression profiles from matched primary and metastatic samples support a nonlinear process for metastasis. A “poor prognosis” genomic signature was similarly found in both primary and matched metastases [12,13,14]. The findings suggest that cells released early from a small primary tumor may already have the potential to form metastases [15]. Another study using mouse models of breast cancer, initially reasoned that if the metastatic process is linear, even if dissemination of tumor cells occurs early, it is only the genetically-progressed cancer cells that would have metastatic capabilities [16]. However, comparative genomic hybridization (CGH) profiling from DTCs harvested from the mouse bone marrow were indistinguishable from early harvests (9 weeks) and late harvests (27 weeks). Surprisingly, there was no detectable increase in genomic aberrations from the younger to older animals. This study appears to suggest that metastatic dissemination is not a result of selection of the tumor cells within the tumor. Data from another comparative genomic analysis using a parallel progression model of primary and metastatic cells identified DTCs in the bone marrow of 607 breast cancer patients regardless of stage or primary tumor size [16], these findings support a model where the metastatic process seems to begin almost simultaneously with tumorigenesis. 

## 3. Mechanisms for Tumor Cells to Enter the Vasculature

One reason for the low metastatic efficiency of cancer cells, is that the majority of CTCs are likely pre- or post-apoptotic upon entering the bloodstream [17,18]. Different areas of tumors are likely actively growing and dying simultaneously. Due to poor perfusion and insufficient delivery of oxygen and nutrients, tumors may have a necrotic core and/or necrotic pockets within the full tumor. The recruited vasculature is immature and disorganized with an abnormal or absent basement membrane promoting “leakiness” of the vessels (reviewed in [19]). The tumor vasculature is inadequate to maintain consistently viable tumor cells, and it also acts as a conduit for dying or dead tumor cells to passively enter the bloodstream. Even before the cells enter the inhospitable environment of the bloodstream, the majority is, or will soon be, dead. Of specific importance are the viable cells which enter the vasculature. Basic enumeration studies do not currently distinguish the viable CTCs from those which are not viable, however there are some limited studies that examine CTC viability [20,21,22]. High levels of viable CTCs would predict a poorer prognosis than that from dead CTCs. These cells may passively or actively enter the bloodstream. It is possible that dissociating tumor cells at the leading edge of the tumor are “pushed” into the bloodstream by the division and expansion of the cells behind them [23]. Intravasating tumor cells may also find the abnormal vasculature an easy entryway into the bloodstream. However, cancer cells are also capable of “squeezing” through an endothelial cell layer, then degrading the extracellular matrix and the basement membrane underlying the endothelial cells. Interestingly, in vitro studies examining this active intravasation process show insignificant interruption of the endothelial cell barrier [24]. Detailed mechanisms for how the tumor cells migrate through or between the tight junctions of the endothelial cells remains unknown. In many cases, in order for tumor cells to migrate and invade, they undergo an Epithelial to Mesenchymal Transition (EMT) [25,26,27,28]. EMT is a developmental program normally employed during embryogenesis and during the healing of epithelial tissues in adults. Carcinoma cells can inappropriately utilize this process to gain certain mesenchymal features while losing some epithelial traits, promoting increased malignant traits such as increased migration and invasion. In line with the idea that metastatic progression is not linear, some studies have shown that cells can acquire EMT traits to disseminate early, even when lesions are preneoplastic [16,29]. While a large body of evidence supports carcinoma cells undergoing EMT programs in breast, colorectal, ovarian, pancreatic, prostate, renal and other cancers [30,31,32,33,34,35,36,37], the cells do not make a complete switch from all epithelial to all mesenchymal characteristics. Even more studies have begun to highlight the likelihood of a partial EMT in which tumor cells retain the appropriate levels of epithelial qualities while acquiring some mesenchymal properties for optimal metastatic potential [26,38,39,40,41,42,43,44,45,46,47]. 

A shift toward a more mesenchymal phenotype will affect CTC collection efficiency. Currently, the only FDA approved method for CTC collection is the CellSearch system which utilizes epithelial cell markers for identification, but tumor cells may downregulate or lose these markers before or during dissemination [22]. Other methods of CTC isolation are label-free techniques utilizing filtration and fluid dynamics based on tumor cells size and deformability and not on epithelial marker detection. Different CTC collection and isolation techniques have been extensively reviewed, and thus will not be discussed here (reviewed in [48,49,50,51,52,53,54]). However, it is important to recognize that all studies discussed in this review did not use the same CTC isolation method and any incompatible results may be attributed to different CTC isolation approaches, and a component of variability is likely due to differences in detection method.

Some tumor cells may have the ability to migrate collectively. In such cases, the tumors retain E-cadherin expression indicative of an epithelial phenotype which allows for the cells to remain attached via cell-cell junction [55,56]. Tumor cells may also invade into the vasculature with macrophages as partners [57,58,59]. Macrophages and tumor cells alternate in single file, and it is suggested that the macrophages secrete proteinases to promote the tumor cells, deficient in overactive proteinase production, to invade. Even if CTCs intravasate as single cells, some can activate and cause aggregation of platelets leading to the formation of tumor cell-induced platelet aggregations. These aggregations enhance CTC survival by protecting them from immune attacks and enhance small vessel trapping [60]. Tumor-derived microemboli may also be released into the bloodstream [61,62,63], and CTC clusters, ranging from 2–50 cells, have been detected in the circulation of metastatic cancer patients [61,63,64,65,66]. Although CTC clusters are rare, they have a 23- to 50-fold increase in metastatic potential [67] for a multitude of reasons. Clumps of cells have more protection from sheer stress, may be better hidden from immune surveillance, and larger emboli can more efficiently trap in smaller vessels as the diameter decreases [9,61,68].

Metastasis requires the dissemination of primary tumor cells, and passage via the bloodstream (i.e., hematogeneous) allows for an extensive dissemination. However, it should be noted that some metastases may not arise from CTCs since some tumor cells have been shown to migrate along nerves [69,70,71] or along endothelial cells [72].

## 4. Current Anti-Tumor Therapies may Inadvertently Increase CTCs

**Figure 2:**
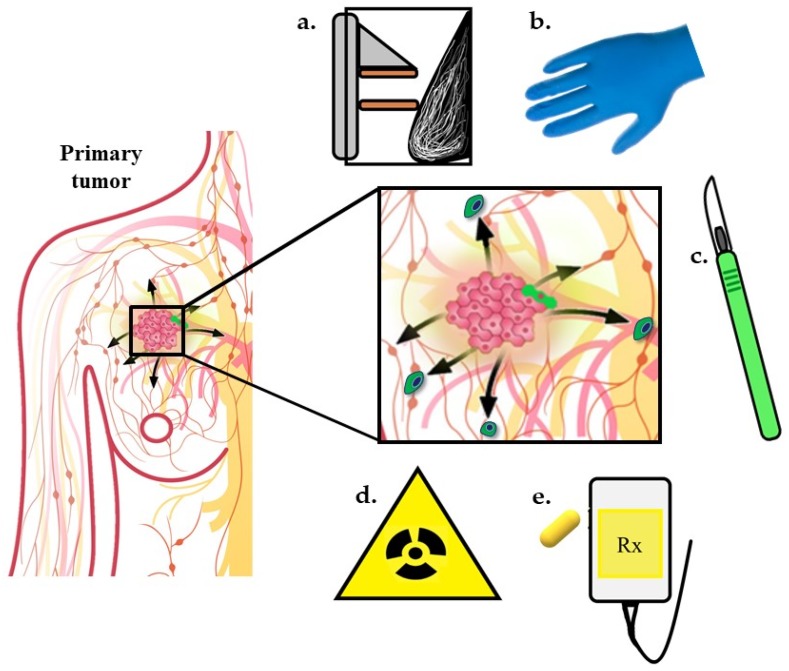
All current treatment modalities have the potential to promote CTCs. Early studies have shown that pressure such as that from mammograms (**a**) and tumor palpations (**b**) may promote tumor cell release. Surgical procedures (**c**) including biopsies and complete tumor resections can promote CTCs. Other possible primary tumor curative treatments such as radiotherapy (**d**) and chemotherapy (**e**) may inadvertently increase CTCs. Figure adapted from nationalbreastcancer.org [85].

Surgery, radiotherapy, and/or chemotherapy are current standards of care for locoregional disease. Unfortunately, some patients develop distant metastases either prior to diagnosis or despite initial treatment of local disease. One reasonable explanation is that the metastases are already present at the time of initial diagnosis and therapy, but below our thresholds of detection, and over time, eventually grow to macroscopic masses. However, accumulating evidence suggests the treatments to control locoregional disease may, in some cases, promote metastasis [48,73] (Figure 2). CTC collection and enumeration studies have shown CTC numbers correlate with disease progression and metastasis in different human cancers where increasing numbers are predictive of lower PFS and OS [74,75,76,77,78,79,80,81,82,83,84], but it is unknown whether current treatments are directly influencing CTCs or whether CTCs released during therapy are capable of forming overt metastases. Preliminary studies have shown all cancer treatment modalities have a potential to increase CTCs. These studies are preliminary, and in some cases, use small sample sizes and therefore statistical significance remains to be determined. Below is a summary of preclinical and clinical data in which treatments necessary for cancer control may increase CTCs in some patients. 

### 4.1. Surgical Procedures

In order to determine whether surgery directly affects the dissemination of tumor cells within the vasculature, a baseline of CTCs would need to be established relatively close but prior to surgery and blood containing CTCs would need to be collected at the time of surgery. Collection of CTCs at a single time point 24 h post-surgery (likely even an hour after surgery) will not accurately determine the effect of mechanical manipulation of surgery due to CTC clearance. The process of CTC clearance can be from successful dissemination or, more commonly, due to death. On the other hand, waiting too long (likely longer than a few days) may also yield inaccurate results. Measuring CTCs at longer time points post-surgery may be complicated by new CTCs released from metastatic or unresected sites induced to proliferate upon primary tumor removal [86]. Instead, there have only been a few published studies on perioperative detection of CTCs or following tumor/ organ removal, and most of these have involved low patient numbers summarized below.

An early study from Hansen et al. [87] detected tumors cells in blood collected intraoperatively in 57 of 61 patients with cancer who underwent surgery for an abdominal, orthopedic, urological, gynecological, or head and neck malignant tumors. Unfortunately, the study did not include collecting blood prior to surgery to determine whether CTCs increased as a direct result of oncologic surgery, so alterations to CTCs could not be assessed. More recent studies have been performed to specifically answer whether surgical procedures promote increases in CTCs. For example, blood samples were collected from before and during lung cancer surgery, and CTCs were quantified and compared in both conditions [88]. The blood collected in this study was from the pulmonary vein of 30 patients undergoing lobectomies and open thoracotomies. Before surgical manipulation, CTCs were detected in a majority of patients and at the time of completion of the lobectomy, CTC counts significantly increased. A separate study examining 20 patients with colorectal cancer with already established liver metastases reported that 50% of cases had an increase in CTCs during hepatic lesion resection [89]. One study examining CTCs in patients with non-small cell lung cancer (NSCLC), collected blood from each patient via the radial artery while the patients were under anesthesia. Blood was collected just before, during, and just after pulmonary vein dissection. Four of 16 patients originally negative for CTCs before surgery were positive following pulmonary vein dissection. The authors conclude that tumor cells can be dislodged and detected after lung cancer surgery [90]. A larger study including 139 patients with hepatocellular carcinoma compared pre- and post-operative CTC numbers [91]. Blood was harvested 1 day prior to surgery and immediately after surgery. Compared with the preoperative CTC counts, the postoperative CTC counts increased in 58 (41.7%) patients. It was concluded that surgical liver resection is associated with an increase in CTC counts. It should be noted that post-surgery, 35 (25.2%) patients had a decrease in CTCs and the CTC number did not change in 46 (33.1%) patients. Yet another recent study collected blood samples from the peripheral artery just before and immediately after partial or radical nephrectomy [92] and compared CTC numbers between a laparoscopic or open surgical approach. Patients underwent laparoscopic partial or radical nephrectomy or open partial or radical nephrectomy. Open radical nephrectomy resulted in a significant increased number of CTC immediately post-surgery with no significant differences observed between the other three procedures. Additionally, a higher detection rate of CTC has been demonstrated in the blood of patients with liver, cervical, colorectal cancers following surgical procedures [91,93,94]. Interestingly, there has yet to be a study implicating any surgical procedures relevant in breast cancer removal. As part of a study from Li et al. [95], endoscopic and/or open radical mastectomy performed on 110 female patients with breast cancer showed a trend in increasing tumor cells from pre-surgical CTCs to post-surgical CTCs, but it did not reach significance. One limitation of this breast cancer study was the post-surgical blood draw occurring 12 h after surgery, allowing ample time CTC clearance.

Besides surgery, other lesser procedures have the ability to disrupt tumors and increase CTCs. Simple pressure which occurs during palpation can increase CTCs immediately. Using an in vivo photoacoustic and fluorescent flow cytometry technique to monitor CTC release in real time from mice transplanted with melanoma cells, a weight was used to approximate the pressure of palpation and applied to melanoma tumors on the backs of mice [96]. CTCs increased during the time of the applied pressure and remained high for an hour after the pressure ended, after which, CTC number began to decline. Similar results were observed by squeezing the tumors with fingers, and CTCs were not detected using weights lower than 50g. Juratli et al. [97] tried to replicate the results using breast cancer cells (MDA-MB-231) inoculated into a mammary gland. When the tumors approximated 50mm^3^ (2 weeks), a weight (400g/0.5cm^2^) was placed atop to mimic the pressure from a mammogram. While there was a minor increase in CTCs detected while the tumor was under pressure, it did not reach significance. The same group also monitored CTC release after incisional biopsy, punch biopsy, and complete resection in their mouse models. During a 15-minute incisional biopsies of melanoma tumors, CTCs drastically increased and remained high for the first hour afterwards. After the first hour, the CTC rate lowered but was still significantly elevated above the rate from before biopsy and remained elevated until the end of the experiment (>2 h) [96]. Punch biopsies of the mammary tumors increase CTCs which remained high for 2 h after the biopsy [97]. Interestingly, no CTCs were observed during resection of the melanoma or mammary tumors or 2 hours post-surgery. Clinically, incisional biopsy has been shown to increase CTCs in 4 of 25 patients [98] and 2 of 10 patients [99] with oral squamous cell carcinoma. 

Finally, fine or core needle biopsies are critical to diagnosing multiple cancers. Multiple studies have shown tumor cells have the ability to seed along the needle track [100], but do these types of biopsies also promote CTCs? Mathenge et al. [101] begin to answer this question using a mouse model of breast cancer and harvesting blood after needle biopsy. Six hours after needle biopsy, a significant increase in CTCs are detected. After needle biopsy, mammary tumors were removed from the mice which had their tumors biopsied and controls which did not. One week after tumor removal, all mice were sacrificed, and lungs were analyzed for metastasis. Mice which underwent biopsies harbored significantly greater lung metastases suggesting that the biopsies may unintentionally promote metastasis by causing CTC shedding. Of note, this study employed the highly aggressive metastatic murine breast cancer line, 4T1. These cells are primed for both metastatic dissemination and growth, and likely have multiple mechanisms to endure the stress of circulation, reattach, disseminate, and regrow in distant tissues. This does not mean all additional CTCs produced by biopsy will metastasize since metastasis is a very inefficient process. Furthermore, it has yet to be determined if any CTCs released by surgery or other mechanical manipulations are even viable. However, another study examines the use of transrectal ultrasound-guided biopsy (TRUS), a standard procedure used for prostate cancer diagnosis [102]. Importantly, blood collection for CTC analysis was completed before and 30 min after prostate biopsy in 115 men with elevated PSA levels. Multiple (8–12) tissue core biopsies are required for the detection of prostate cancer, as it is a multifocal disease, and these biopsies were associated with a significant increase in bloodborne prostate tumor cells in men with histologically confirmed cancer. No biopsy-related change was detected in the men without cancer, demonstrating that the normal gland will not shed cells during TRUS biopsy. The patients for whom increased CTC counts were observed, had a shorter progression-free survival, implying faster disease progression, independently from Gleason score, biopsy core positivity, and presence of CTCs at baseline. Since patients did not have clinically evident metastasis at primary diagnosis, it is unlikely the CTCs detected in this study arose from an alternative source besides the primary lesion. It is thus concluded that the mechanical trauma from TRUS led to the increase in CTCs, which ultimately affected disease propagation [102].

### 4.2. Radiotherapy 

Early experiments using animal models have indicated that multiple tumor types irradiated with low, non-curative doses of radiation, insufficient for local control, are associated with higher risk of metastasis [103,104,105]. Interestingly, all metastases were localized to the lungs and in the Kaplan et al. [104] study, the pulmonary metastases grew in the arteries and arterioles, implicating possible vascular transit from the primary tumor site. Another mouse model using Lewis Lung carcinoma cells grown in the hindlimbs of mice utilized radiation of the implanted tumors to accomplish local disease control. However, the irradiated mice had an increased rate of lung metastases compared to the non-irradiated mice [106]. A more recent study showed an increase in CTCs from tumors of subcutaneously implanted 4T1 cells in the left rear limb at the first 10 and 20 min after a 12 Gy dose [107] Although CTCs originally increased, there was no differences in CTC numbers between the irradiated mice and the control mice at 8 hours, and irradiation did not correlate with an increase in metastases. The authors reasoned that the lack of metastatic tumor burden following release of CTCs after high dose radiation therapy indicates low CTC viability, clonogenicity, or both. 

Studies indicate that radiotherapy (RT) can alter tumor cell biology to make them more aggressive than non-irradiated cells [108,109,110,111,112,113,114,115]. In addition to altering cancer cell phenotype, radiation may promote the release of cells into the circulation due to radiation-induced structural damage to blood vessels within the tumors. Furthermore, radiation can induce changes in global signaling such as alterations to cytokines. Granulocyte-macrophage colony stimulating factor (GM-CSF) induced upon irradiation is one cytokine that stimulates tumor self-seeding [116]. “Tumor self-seeding,” where CTCs in the blood can re-colonize back at the original site has been documented in breast cancer, colon cancer, and melanoma tumors in mice resulting in accelerated tumor and angiogenesis. It has been reported that about half of the breast cancer patients after RT, have recurrence disease at the same site [117]. Although this tumor recurrence is not entirely due to CTCs, these observations suggest that CTCs play dual roles in tumor dissemination and tumor recurrence after radiation. There is some evidence to support radiation promoting metastasis, however there are extremely limited studies aimed to determine whether RT directly promotes tumor cell release. Martin et al. [118] sought to determine whether radiotherapy could mobilize viable NSCLC cells into patients’ circulation. Of the 27 initial patients with locoregional advanced or metastatic disease, 7 of 9 treated with palliative RT had increased CTCs 24 h after the first RT fraction compared to their pretreatment baseline. As previously mentioned, CTCs can clear within minutes after release into the bloodstream. Future studies of CTC counts following radiation at time points within the first 24 h of RT are of interest. However, differences were detected and perhaps tumor cells continue to be shed hours after RT. For another 4 of 8 patients treated with curative-intent RT, CTCs also increased. Collected CTCs exhibited elevated γ-H2AX levels consistent with exposure to high doses of ionizing radiation which indicated that they originated from within an RT-treated tumor. The CTCs isolated in this study were proven to be viable as they were positive for Ki67 staining, a marker of proliferation, and the ability to propagate in vitro. The authors conclude that perhaps RT is successful because of high cumulative dose of radiation, but in the early stages of radiotherapy, up to one half of irradiated tumor cells may escape to the circulation. The authors also noted the presence of CTC clusters collected in some patients after RT, even though these clusters were initially absent in the same patient during initial blood draw. The idea that RT may induce the release of small pieces of the tumor into the vasculature is of additional concern as CTC clusters (2–50 cells) have been shown to have increased metastatic potential by 23–50 fold [67]. Conversely, another pilot study concluded that RT reduced CTC counts [119]. Here, blood was collected from patients with localized NSCLC before and one-half to two-thirds through each patient’s RT course. In 14 of the 15 patients, CTC counts dropped to below the detectable threshold. The exception was one patient who was found to have developed metastatic disease soon after RT completion. The opposing outcomes in the above studies may be explained by the fact that one group specifically examined the effects of RT in more advanced NSCLC compared to the other. These conflicting findings enhance the need to better understand the biology underlying CTCs and the impact of timing cancer treatments.

While we still need to determine whether radiotherapy increases CTCs, if the CTCs are viable, and if it is the initial release of CTCs that ultimately promote metastases, a meta-analysis completed using 2 large cohorts of early breast cancer patients concluded that radiotherapy results in positive patient outcomes [120]. In the first cohort (1697 patients), the 4-year OS for CTC- positive patients who received RT was 94.9% while those who did not receive RT was 88.0%. RT did not affect the 4-year OS for the CTC-negative group (RT treated non-CTC patients = 93.9% vs non-treated non-CTC patients = 93.4%). RT was also associated with longer OS for the CTC-positive patients, but not in the patients without CTCs. Examining a second cohort (1516 patients), the 5-year DFS was better for patients treated with RT, with or without CTCs at time of treatment (88.0% for CTC-positive with RT vs. 75.2% CTC-positive without RT; 92.3% CTC-negative with RT vs. 88.3% CTC-negative without RT). In this second cohort, RT was also associated with longer local recurrence free survival, DFS, and OS. There are conflicting results between these large cohorts. We need a better understanding of CTC biology and how radiotherapy may affect CTCs. Perhaps determining molecular profiles of CTCs and/or tumors will aid in determining the susceptibility of individual’s disease to radiotherapy. In any case, additional studies with more patients with different types of cancers and different doses of radiation will need to be completed to make any conclusions about CTC release, metastasis, and overall survival.

### 4.3. Chemotherapy 

CTC reduction during and after courses of different chemotherapies is associated with a favorable treatment response and improved survival [121,122,123,124,125], while an increase may indicate loss of therapeutic benefit [74,75,126]. However, as in the case with the other treatment modalities, some chemotherapies have been linked to an increase in metastasis. For example, angiogenic inhibitors have not been as effective for improving the survival of patients [127]. Animal models have shown that although angiogenic inhibitors are successful in reducing the primary tumor [122,123], inhibitors such as sunitinib, sorafenib, and SU10944, can induce hypoxia in the residual tumor promoting invasion and metastasis. Another targeted therapy that has been shown to induce metastases in animals is BRAF inhibitors. While vermurafenib treated tumors originating from implanted human melanoma cells were growth inhibited, the vermurafenib-sensitive cells secreted factors which promoted the growth and metastases of vermurafenib-resistant tumors [128]. PLX4720, another BRAF inhibitor, increased lung, liver, and kidney metastasis of RAS mutant melanoma in mice. Although androgen deprivation therapies have been shown to reduce prostate cancer size, they too may lead to increased metastases in some patients [129], as both Casodex (bicalutamide) and MDV3100 (enzalutamide) were shown to also increase metastasis in mice. Paclitaxel was shown to promote breast cancer metastasis by increasing local and systemic inflammation, expansion of tumor vascular networks, thus increasing lymphatic metastasis of TLR4-positive tumors [130]. Everolimus, an mTOR inhibitor, increased the occurrence of distant metastases in a rat model of pancreatic cancer [131], and cyclophosphamide has been shown to promote metastasis under certain conditions [132,133,134,135]. 

A possible explanation for the increase in metastases as a result postoperative or adjuvant chemotherapy is that some chemotherapies may render distant tissues more prone to metastatic seeding as a result of a systematic release of cytokines [136,137]. Another possible explanation is that chemotherapies may inadvertently promote CTC release, or changes in the population of CTCs released, which in turn may promote or exacerbate metastasis. There are few studies examining whether increases in CTCs and/or changes in CTC biology could possibly be a direct result of therapy. An analysis of CTCs from 10 breast cancer patients pre- and post-treatment was completed [66], and in the 5 patients who responded to treatment, there was a decrease in total CTCs, but also a shift in EMT characteristics of the CTCs detected. The remaining CTCs detected after treatment were less mesenchymal when characterized with commonly used markers. In contrast, the 5 patients whose disease progressed while on therapy had an increase number in the mesenchymal CTCs. In this study, one patient initially responded to therapy, developed resistance, and then transiently responded to treatment. CTCs were monitored throughout this process and more mesenchymal CTCs tracked with nonresponse. Initially, the mesenchymal CTCs dropped and predominantly switched to CTCs with more epithelial markers, but upon disease progression, the CTCs switched back, presenting more mesenchymal markers. While this study did not answer whether chemotherapy directly promotes CTCs, it did begin to highlight the presence of different cellular markers and differing populations of CTCs, implicating the role of EMT in human breast cancer specimens. A different group examining another set of patients (27) with invasive breast cancer revealed that chemotherapy caused significant changes in CTCs collected [138]. Some patients experienced an increase in total CTCs as well as an increase in EpCAM^−^CD45^−^CD44^+^CD24^−^N-cadherin^+/−^ cells, but no changes in the EpCAM^+^CD45^−^CD44^+/−^CD24^−^N-cadherin^+/−^ population. The only major differences in the populations is the presence or absence of EpCAM, the absence of which indicates a shift in CTCs from epithelial to more mesenchymal phenotype. Similarly, a third study examined CTCs from 62 metastatic breast cancer patients before and after chemotherapy [139]. The nonresponders to chemotherapy had an increase in CTCs with a cancer stem-like phenotype and partial EMT, suggesting the type of CTC detected in this study is resistant to the therapy. It would appear from these preliminary studies, chemotherapy may not induce CTC release, but rather select for more dangerous tumor cells (i.e., partial EMT and/or more cancer stem-like) potentially resistant to chemotherapy. Along similar lines, studies from our group have shown that chemotherapy [140], as well as cells which have undergone a partial EMT [141,142,143] or exhibit more stem-like character, have an enhanced ability to reattach after suspension via novel tubulin-driven membrane protrusions termed microtentacles, a possible mechanism for increased metastases.

Studies directly examining the effect of chemotherapy on mobilization of CTCs are scarce. However, we have identified two studies which concluded chemotherapies can induce CTC release into the bloodstream. Micro-anatomically structures named tumor microenvironment of metastasis (TMEM) within the tumors have been shown to aid in tumor cell intravasation [144,145], and their presence has been associated with murine mammary tumor and human breast cancer metastasis [146,147]. The three components of a TMEM are a perivascular macrophage, an endothelial cell, and a tumor cell expressing invasive isoforms of the actin-regulatory protein MENA in close proximity to the endothelial cell. Although randomized prospective studies demonstrating that the neoadjuvant use of paclitaxel therapy increased the rate of complete pathological response, it did not improve overall survival [148,149]. Since paclitaxel promotes the influx of macrophages into the primary tumor and these cells are required for TMEM assembly [150,151], Karagiannis et al. [152] hypothesized that paclitaxel treatment may increase TMEM sites, leading to an induction of CTCs. Using intravital imaging of mouse mammary tumors in murine models, patient derived xenografts, and fixed human breast cancer tissue, they were able to elucidate mechanisms by which paclitaxel may enhance the dissemination of CTCs. First, paclitaxel increases the infiltration of TIE2^hi^/VEGF^hi^ macrophages promoting the formation of more TMEM sites. Second, paclitaxel increases the frequency of functional TMEM sites where increased vascular permeability is achieved by transient vessel “bursting.” Third, it was determined that paclitaxel induced expression of the more invasive MENA isoforms resulting in an increase in tumor cells invasion. In all models examined, paclitaxel treatment increased the number of CTCs by approximately 2-fold. Further examination of the lungs revealed an increased incidence and number of micrometastases in the treated mice, likely as a result of the increase in CTCs. Additional studies into the effect of chemotherapies on TMEMs revealed a doxorubicin/cyclophosphamide combination therapy affects TMEM density, TMEM activity, and CTCs similar to paclitaxel. Chang et al. [153] were unable to verify an increased infiltration of macrophages (TIE2^+^) within the tumors in their model, but instead showed that the isolated infiltrating macrophages from mice treated with paclitaxel simulated cancer cell invasion more efficiently than the tumor infiltrated macrophages isolated from the untreated mice tumors. They confirm that paclitaxel increases TMEM density and CTCs and established that effect of paclitaxel *in vivo* are due to host-*Aft3* status. Wild-type mice had more CTCs than the *Atf3*-KO mice which was exacerbated by paclitaxel in the WT mice but not in the *Atf3*-KO mice. The complex effect specific chemotherapeutics may play in modulating CTCs still needs to be studied more extensively, but these preliminary studies suggest that the global intervention of chemotherapy may have unintended effects.

## 5. Potential Therapies to Reduce CTCs

Inhibiting cancer progression by preventing dissemination and the emergence of overt metastases is not a new concept. To prevent regrowth at the primary site or growth of overt metastases, adjuvant chemotherapy may be prescribed. For example, targeted therapies such as tamoxifen and aromatase inhibitors are given to breast cancer patients to prevent or delay relapse even when no disease is detectable. The goal of these therapies is to kill or arrest disseminated cancer [154]. As our understanding of CTC biology and detection methods (i.e., liquid biopsies) for CTCs advance, it is plausible to develop agents specifically targeted to CTC destruction. Since CTCs are suspended in the bloodstream, it can be imagined that these cells are undergoing a multidirectional wound response in which they can experience imbalances between microtubule extension and actin contraction [142,155]. Our group has demonstrated that detached cancer cells promote dynamic tubulin-driven protrusions, termed microtentacles, which aid in endothelial cells attachment [140,141,143,156,157,158,159,160,161]. It is hypothesized that repurposing cytoskeletal targeted drugs, frequently used to inhibit growth, and/or developing drug which more specifically target tubulin post-translational modifications, may inhibit microtentacles [155]. Reducing microtentacle formation may reduce initial endothelial cell engagement promoting CTCs to remain in the bloodstream and die by shear stress or fragmentation (Figure 1). Another strategy would be to target the tumor-associated macrophages, which cause transient permeability to the vasculature [150], to reduce the number of CTCs. Karagiannis at al. used rebastanib, a TIE2 inhibitor, to target TMEM-associated macrophages. While rebastanib treatment did not affect the overall TMEM number or density of tumor infiltrating macrophages, it did significantly decrease the number of CTCs, indicating that TMEM activity was inhibited [152]. Once in the bloodstream, CTCs may bind to platelets to avoid leukocyte attack [162,163] and the CTC-platelet aggregates may release cytokines which can attract granulocytes [164]. Targeting the CTC-platelet-granulocyte interaction may also lead to the reduction in CTCs [164,165]. Finally, Gkountela et al. have identified six FDA-approved compounds with the ability to reduce CTC clustering ability and suppress spontaneous metastasis in xenograft models [166]. 

## 6. Conclusions 

Importantly, none of the current treatments should be abandoned since the prognostic benefit of these procedures and therapies still strongly outweigh their possible negative effects. Future studies are needed to determine what extent surgical techniques and manipulation, radiotherapy, and chemotherapy have on CTC shedding, biology, and cancer recurrence rates. With approximately 90% of cancer patients dying from metastatic disease, we are hopefully experiencing a shift in cancer research to preventing and eradicating metastasis [1,167,168]. As the ability to detect and characterize CTCs from patients rapidly evolves, we believe that more efforts should be dedicated to understanding the effects of already established interventions and new treatment strategies on CTC number and metastatic efficiency. As the frontier of CTC research continues to expand, the complex underlying biology involved in the metastatic cascade will be clarified. We hope that with these clarifications will come new therapeutics which effectively target CTCs and drivers of metastasis, leading to additional improvements in patient outcomes.
